# Contemporary outcomes of open repair of thoracoabdominal aortic aneurysm in young patients

**DOI:** 10.1186/s13019-014-0195-4

**Published:** 2014-12-10

**Authors:** Neil Johns, Russell W Jamieson, Carlo Ceresa, Carl Moores, Alastair F Nimmo, Orwa Falah, Paul J Burns, Roderick TA Chalmers

**Affiliations:** Department of Vascular Surgery, Royal Infirmary of Edinburgh, Edinburgh, EH16 4SB UK

**Keywords:** Thoracoabdominal, Aorta, Aneurysm, Young

## Abstract

**Background:**

Endovascular technology now permits total endovascular thoracoabdominal aortic aneurysm (TAAA) repair with high volume centres reporting encouraging results. The long-term durability of such stent grafts is unknown, leading to concerns regarding their use in younger patients. This study reports contemporary outcomes of open repair in young patients.

**Methods:**

Outcomes for patients age 60 or younger undergoing open TAAA repair between June 1999 and August 2013 with prospective collected data were analysed retrospectively.

**Results:**

Thirty-seven patients (31 men, 84%) with a median age of 56 (range 22–60) were identified with a median TAAA diameter of 6.9 cm (range 5.6-11). Aneurysm aetiology included degenerative change (18), dilation of chronic dissection (10), connective tissue disease (7) and mycotic degeneration (2). Crawford Type IV TAAA were most commonly treated (17), followed by Type II (10), Type III (7) and Type I (3). Two (5%) patients died in hospital, one from multiple organ failure and one from respiratory failure. Three patients (8%) developed temporary paraplegia, all of whom made a complete recovery and 4 (11%) patients required temporary renal replacement therapy. Median critical care stay was 5 days (range 2–28) with an in-hospital stay of 14 days (range 7–83). During a median follow-up of 72 months (range 13–171), no patient subsequently required any further aneurysm related surgical or radiological intervention. The mean (SEM) survival time was 138.5 (11) months. The 5 year survival was 79.7% (8.3) including early deaths, with no aneurysm related complications.

**Conclusions:**

The outcome of open TAAA repair in patients aged less than 60 years is favorable. It is against these results that evolving endovascular interventions must be compared.

**Electronic supplementary material:**

The online version of this article (doi:10.1186/s13019-014-0195-4) contains supplementary material, which is available to authorized users.

## Background

The incidence of thoracoabdominal aortic aneurysm (TAAA) is increasing with the condition more common in the elderly population but around 15% of patients are under the age of 65 [1]. Endovascular techniques offer a minimally invasive alternative to open surgery and may be combined with the open procedure in hybrid operations for aneurysms not suitable for an entirely endovascular approach [2]-[5]. Total endovascular repair remains challenging but increasingly good results have been reported by large specialist centres [6]-[9]. The long-term durability of such stent grafts is, however, unknown, raising concerns regarding their use in younger patients. Endovascular enthusiasts measure their results against open surgical repair but little contemporary data are available specifically reporting outcomes of open surgery in the younger patients. We report our single centre experience of open repair in patients age 60 or under to allow comparison against emerging technologies.

## Methods

Data were collected prospectively in a clinical database. Additional post-discharge information was obtained from hospital and general practice records. All patients undergoing repair of a TAAA aged sixty or under over the 12-year interval from 1998 to 2013 were analysed (Table 1).

**Table 1:** **Patient demographics**

	No of patients (n = 37)
Age (years)*	56 (22–60)
Sex ratio (M : F)	31 : 6
Cardiac disease	12 (32)
Ischaemic heart disease	11 (30)
Arrhythmia	1 (3)
Respiratory disease	6 (15)
COPD	6 (15)
Renal Disease	5 (19)
Biochemical evidence of dysfunction	5 (19)
Diabetes mellitus	1 (3)
Smoking history	31 (84)
Current smoker	16
Ex smoker	15
ASA physical status^*^	3 (2–4)
Type of aneurysm	
I	3 (8)
II	10 (27)
III	7 (19)
IV	17 (46)
Aneurysm diameter (cm)^*^	6.9 (5.6 -11.0)
Pathology	
Chronic dissection	10 (27)
Degeneration without dissection	18 (49)
Connective tissue disease	7 (19)
Mycotic	2 (5)

Values in parentheses are percentages unless indicated otherwise; ^*^values are median (range). COPD, chronic obstructive pulmonary disease; ASA, American Society of Anesthesiologists.

### Preoperative assessment

**Figure 1:**
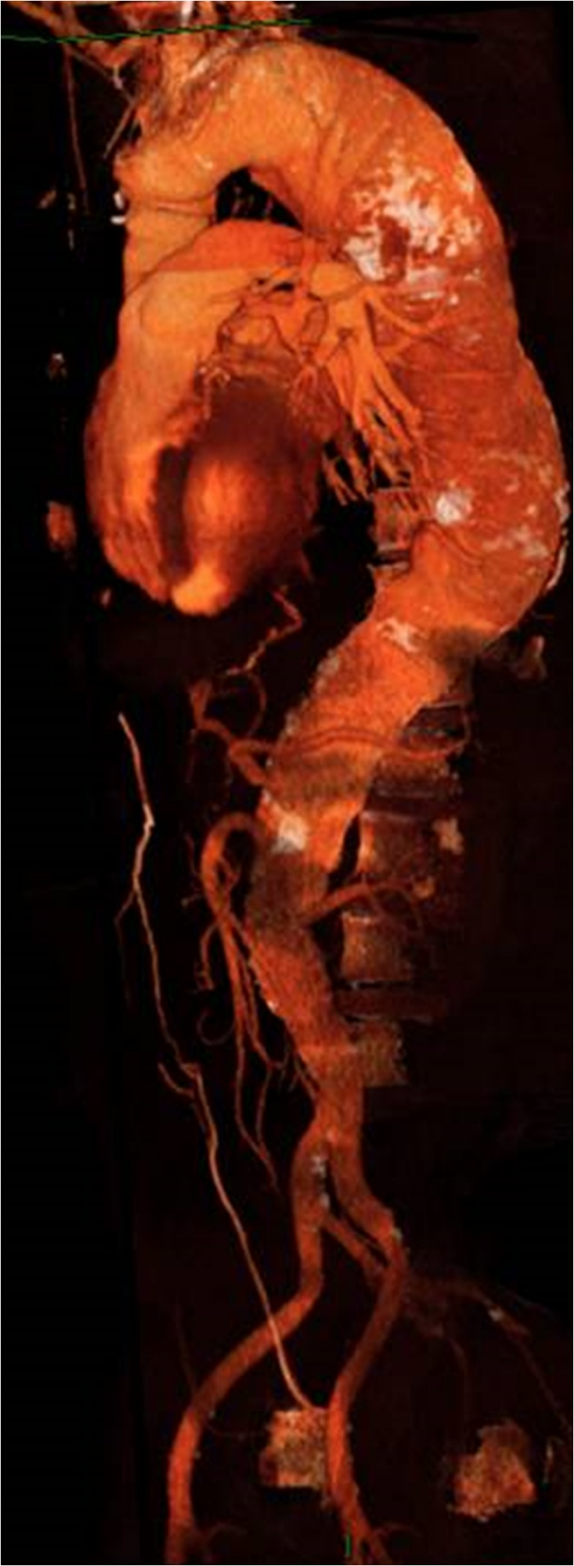
**A CT reconstruction of a type II TAAA.**

A comprehensive preoperative evaluation was undertaken for all patients, including computer tomography angiography (CTA) reconstruction of the aneurysm morphology (Figure 1), resting and stress echocardiography, lung function tests, cardiopulmonary exercise testing and in selective cases CTA of the coronary arteries. Patients were assessed by a multidisciplinary team including Consultant surgeons, dedicated vascular anaesthetists, interventional radiologists and if necessary a Consultant cardiology opinion was sought.

### Operative management

General and epidural anaesthesia were used in all patients in conjunction with passive hypothermia of 32-34**°** C (Type IV) and active cooling on left heart bypass to 32**°** C with Type I, II and III TAAA during the period of visceral ischaemia. Detailed cardiovascular monitoring included pulmonary artery catheterization and/or transoesophageal echocardiography, invasive arterial blood pressure monitoring and central venous pressure monitoring. Strategies to reduce blood loss included cell salvage return transfusion, rapid infusion systems, fibrin sealants and bioglues. Near-patient testing of haemoglobin, arterial blood gases, electrolytes, glucose, lactate and coagulation (ROTEM thromboelastometry; Tem International GmbH, Munich, Germany) was used to provide frequent intra-operative evaluation of these parameters. For all TAAA with the exception of Type IV repairs, a cerebrospinal fluid drain was sited pre-operatively. The drain was opened and set to 10 mm Hg above the level of the atria during and after surgery except during the period of aortic clamping when the drain level was reduced to 0. Motor evoked spinal cord potentials were not recorded. Lower limb movements were assessed hourly after surgery and in the case of weakness the mean arterial pressure was raised and the cerebrospinal pressure lowered in order to improve spinal cord perfusion pressure.

**Figure 2:**
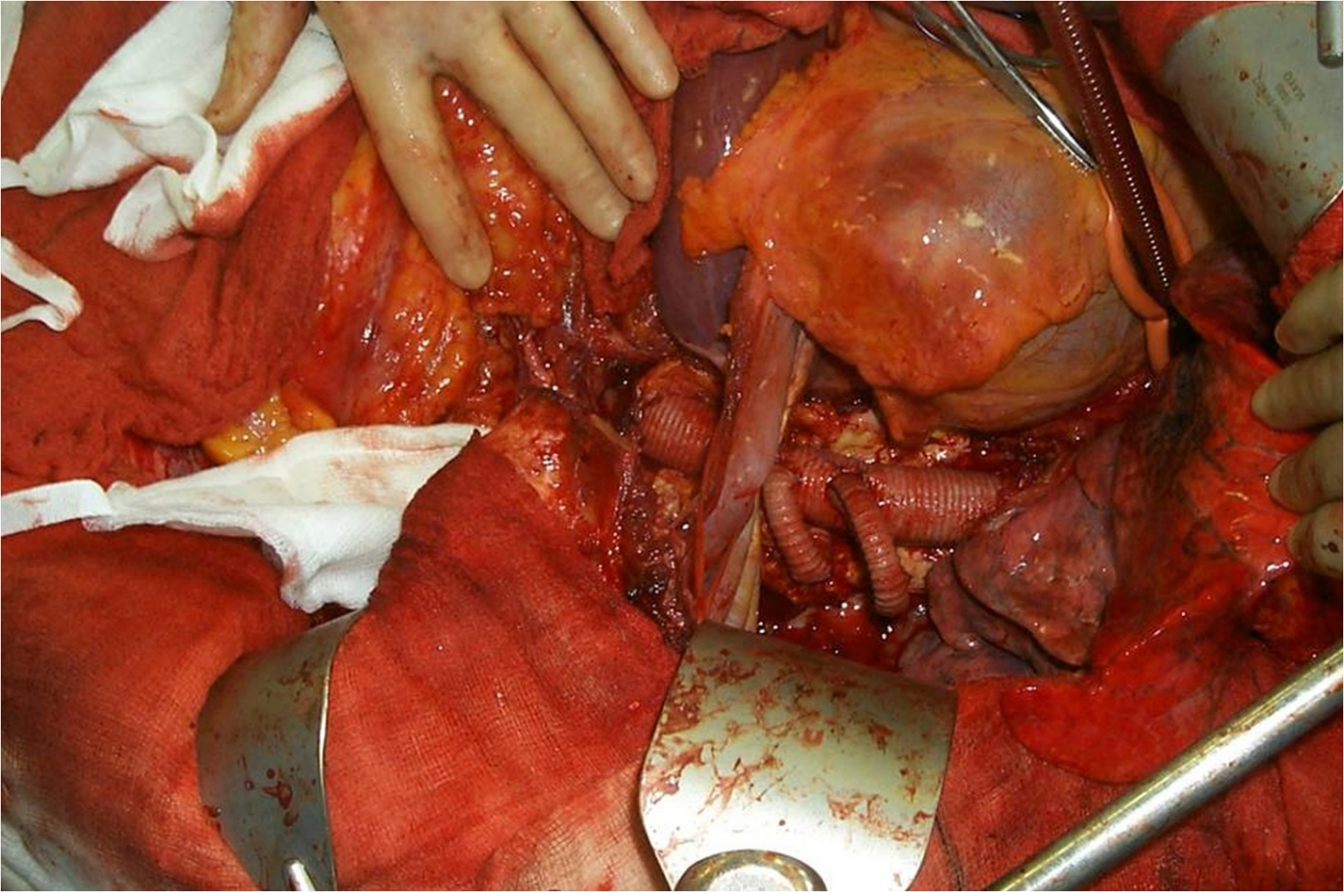
**A complete replacement of the thoracic aorta and 2 jump grafts coming off to revascularise the intercostals arteries.** The L lung is deflated, the LHB cannula is in place.

For Type I, II and III the patient was positioned in a modified right lateral decubitus position with the arms near horizontal and the hips at 30-45**°**. Dual lumen endobronchial intubation allowed deflation of the left lung and a thoraco-laparotomy via the 6^th^ intercostal space extending down the midline of the abdomen afforded good exposure. Left medial visceral rotation was used to expose the abdominal aorta. Left heart bypass was achieved via pulmonary vein and left femoral cannulation and latterly by via a conduit anastomosed end-to-side to provide continuous perfusion to the left leg. The bypass circuit incorporated a centrifugal pump and a heat exchanger and typically provided a flow rate between 1.5 to 2 litres/minute (Figure 2). For Type IV TAAA the patient was positioned supine and a rooftop laparotomy extending to the left flank was used to allow left medial visceral rotation to expose the aorta from above the left crus of the diaphragm down.

Significant intercostal arteries were reattached on a patch or by means of a jump graft and the visceral vessels incorporated in a beveled anastomosis where possible although separate grafts were used to the left renal in particular when the distance between visceral ostia was too great.

### Post-operative care

All Type I, II and III TAAA patients were admitted to intensive care for a period of postoperative ventilation while Type IV patients were extubated in recovery and admitted to a high dependency unit. Cerebrospinal fluid drainage was maintained for 48 hours and if no weakness detected the drain clamped prior to removal 24 hours later. Renal replacement therapy, vasoactive drug use, ventilation, nutrition and antibiotic therapy were guided by the clinical picture and followed standard critical care protocols. Biochemical evidence of renal dysfunction was classified as an abnormal creatinine on base line blood tests. Pre and post-operative creatinine was used to calculate a percentage rise.

### Statistical analysis

Results are expressed as median and interquartile ranges where stated, percentages are also shown. Survival data were calculated by using the Kaplan-Meier method. The program SPSS (version 20, SPSS, Chicago, IL, USA) was used for all the statistical tests.

## Results

**Figure 3:**
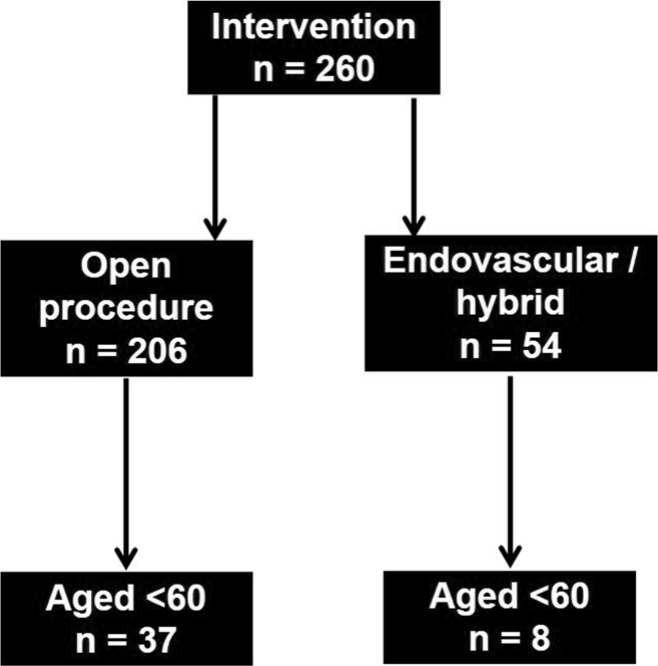
**Flow diagram showing distribution of patients undergoing TAAA repair between 2001 – 2013.**

Thirty-seven patients (31 men, 84%) with a median age of 56 (range 22–60) were identified with a median TAAA diameter of 6.9 cm (range 5.6-11) (Figure 3). This represents 14% of all TAAA repairs performed by our unit over the 12 year time period. Aneurysm aetiology included degenerative change (18), dilation of chronic dissection (10), connective tissue disease (7) and mycotic degeneration (2). Crawford Type IV TAAA were most commonly treated (17), followed by Type II (10), Type III (7) and Type I (3).

The median total duration of surgery was 465 minutes (interquartile range (i.q.r.) 306–613), with a median visceral/renal ischaemia time of 50 minutes (i.q.r. 35–57) and lower limb ischaemia time of 178 minutes (i.q.r. 91–265). A separate graft to one or more of the renal arteries was required in 12 patients (32%) and 10 (27%) had a bifurcated aortic graft. It was possible to remove the endotracheal tube in 26 (70%) of the 37 patients before they left the theatre recovery area, allowing the patient to be transferred to HDU for further management with the remaining 11 patients admitted to intensive care for lung ventilation. The median duration in critical care was 5 days (i.q.r. 2–28) with a total median hospital stay of 14 days (i.q.r. 7–83),

There were two deaths within 30 days giving a mortality of 5.4%. One patient died from multiple organ failure following a type II repair complicated by pneumonia on postoperative day 4. The second, following a type II repair, developed acute respiratory distress syndrome and died on day 13.

Respiratory complications were observed in 6 out of the 11 patients admitted to intensive care for ventilation post-operatively and in 14 patients (38%) in total. Of those extubated immediately after surgery, 2 required non-invasive ventilation and 3 re-intubation and ventilation.

Renal replacement therapy (RRT) was required for 4 patients (11%) with 2 patients having pre-existing renal dysfunction. In 3 cases RRT was required for less than 1 week with the remaining patient on dialysis for 3 months. Ultimately all four patients have remained independent of RRT. A further 10 patients had evidence of post-operative renal dysfunction with a rise in the serum level of creatinine of at least 150% and/or a period of profound oliguria (Table 2).

**Table 2:** **Patient outcomes**

	Crawford type repair		
	I, II, III	IV	All
**Cardiac complications**			
MI/Troponin rise	2 (10)	1 (6)	3 (8)
Arrhythmia	5 (25)	2 (12)	7 (19)
Cardiac arrest	0 (0)	0 (0)	0 (0)
**Respiratory complications**			
Pneumonia	6 (30)	7 (41)	13 (35)
Adult respiratory distress syndrome	1 (5)	1 (6)	2 (5)
Effusion requiring drainage	3 (15)	0 (0)	3 (8)
Reintubation	3 (15)	1 (6)	4 (11)
**Renal complications**			
Creatinine >150% of baseline	5 (25)	5 (29)	10 (27)
Renal replacement therapy	1 (5)	3 (18)	4 (11)
**Neurological complications**			
Temporary paraplegia	3 (15)	0 (0)	3 (8)
Permanent paraplegia	0 (0)	0 (0)	0 (0)
**Reoperation**	1 (5)	0 (0)	1 (3)
**30-day mortality**	2 (10)	0 (0)	2 (5)

Values in parentheses are percentages unless indicated otherwise.

Neurological complications were noted in three patients with two experiencing temporary weakness of the lower limbs**,** which had resolved by the time of the first post-discharge clinic review. The remaining patient had a longer period of paraparesis consistent with a spinal cord ischaemic injury, which resolved over the course of a year (Table 2).

**Figure 4:**
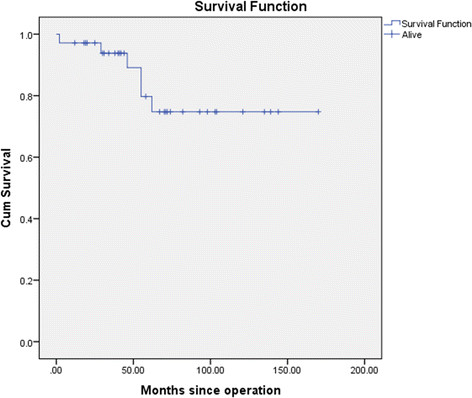
**Kaplan Meier survival plot of patients undergoing TAAA repair between 2001 – 2013.** The mean (SEM) survival time was 138.5 (11) months. The 5 year survival was 79.7% (8.3).

There were 6 deaths in addition to the two 30 day mortalities giving a total of 8. Causes included one pneumonia, one cardiac arrest from an upper gastrointestinal haemorrhage, one urinary tract infection leading to overwhelming sepsis, one myocardial infarction and two unknown due to incomplete records. There were no aneurysm related complications and no patient required re-operation or radiological interventions to the repair.The mean (SEM) survival time was 138.5 (11) months. The 5 year survival was 79.7% (8.3) including early deaths, with no aneurysm related complications (Figure 4).

## Discussion

Our data show that open repair of TAAA in younger patients can be undertaken with acceptable levels of morbidity and mortality, and without the need for re-intervention.

The mortality and morbidity associated with open TAAA repair in specialist centres has been widely reported [9]-[12]. Endovascular enthusiasts, with the caveat that they represent the best possible outcomes and are not applicable to smaller centres, often reference such results. While there is undoubtedly a volume/outcome relationship for TAAA surgery [13] the main concern with using such data to compare endovascular interventions in younger patients is the elderly patient cohort included in these studies. These results are for all age groups and in a contemporary study the median age was 66 years with an interquartile range from 57 to 73 [12]. The very favourable results experienced in our centre compare well against high volume specialist centres’ unselected age group results and demonstrate that in younger patients a much higher level of success can be expected, even in an intermediate volume hospital. Thus when considering endovascular techniques in younger patients comparison against unselected open results gives a false sense of security.

The alternatives to open repair are hybrid repair and branched endovascular stent grafting. The hybrid repair combines both open and endovascular techniques either in a simultaneous or two-staged procedure. Typically a thoracic endovascular stent graft is positioned to exclude the thoracic component with the distal landing zone extended below the visceral aorta by means of open surgical revascularisation of the visceral and renal vessels. This avoids the need for thoracotomy, potential paralysis of the left hemi-diaphragm, and cross-clamping of the aorta in the chest, which, at least in theory, has the potential to decrease overall morbidity and mortality [14]. Despite this a recent review cited a 10.4% 30-day mortality for elective hybrid cases [15], although these results are on an unselected older patient group. While the potential benefit of reduced spinal cord ischemia has been suggested [2], significant rates of cord ischaemia continue to be reported. Proponents of the technique have suggested that staging interventions significantly reduces the risk of spinal cord ischaemia [16] but a recent meta-analysis raises doubts about the significance of the effect reported [17]. Of particular relevance to younger patients, the hybrid repair has been largely advocated as approach best suited for older patients not fit enough for standard open surgery. While it is reasonable to assume that much better results could be expected in younger patients concerns then arise regarding graft thrombosis which has been reported as high as 10% at a short follow-up [14] and questionable durability of these reconstructions in younger people.

The remaining alternative to open surgery is total endovascular TAAA repair. This technique, first reported by Chuter et al. [18], has become increasing popular in specialist centres [6]-[9] which have reported impressive results. In general this technique has been pioneered in patients deemed unsuitable for open surgery. This is best seen when looking at a US national database reporting TAAA repairs in which the number of open repairs has remained static and the number of endovascular repairs has increased significantly [19]. This suggests that centres are attempting endovascular repair in patients who were not considered candidates for conventional open surgery. The technical success reported by skilled endovascular surgeons is very high, even when the surgery is not performed within one specialist unit, but patient selection remains the critical issue with the dangers of visceral and spinal cord ischaemia and endoleaks remaining [20]. Proponents of endovascular repair will argue that in younger patients endovascular results can be expected to be even better that those seen in the frail elderly cohorts that are currently being selected but to date the data to support this argument do not exist. Eight patients underwent endovascular stent grafting during the study period. Long term durability of endovascular repair in young patients is still questionable. Our centre has limited experience of endovascular repair of complex TAAA and with a proven record of success in open surgery reserve endovascular repair for those felt to have an unacceptably high open surgical risk. Such patients typically have major pre-existing co morbidities (predominantly respiratory or cardiac disease) and often have had a previous thoracotomy making the operation technically difficult and dangerous.

For the remaining younger patients three main issues arise with endovascular repair, namely, endoleaks, spinal cord ischaemia and radiation exposure. Endoleaks, where there is a failure to obtain or maintain aneurysm exclusion, remain the Achilles heel of endovascular TAAA surgery. With branched TAAA repair the branched main body must be extended to the target vessels, typically with a covered stent lined with a balloon expandable stent and may also need to be extended either proximally or distally with further stent grafts. This layered graft approach risks component separation. Using mathematical modeling Greenberg’s team studied a cohort of 106 patients and found actual separation in 8% but more worryingly the potential for separation in 38% [21]. In younger patients with a longer life expectancy the likelihood of developing an endoleak must be a factor to consider given the risks of late aneurysm rupture and the requirement for re-intervention to treat such endoleaks. Spinal cord ischaemia also remains a worry with total endovascular repair with Chang et al. reporting a 19% risk of cord ischaemia [22]. For younger patients the total lifetime radiation dose associated, not just with the procedure itself, but also the lifelong follow-up and any subsequent interventions, must not be dismissed. Although the total radiation dose to the patient during endovascular aneurysm repair is relatively low [23], the pre and post-operative CTA imaging exposes the patient to significant radiation [24]. The excess risk of radiation induced solid organ malignancy is greatest the 50 to 55 year age group and estimated at between 0.43 and 1.03 [24],[25]. Although many now consider post-operative follow-up with routine CTA unnecessary [26], should complications arise CTA remains the imaging modality of choice.

Pathology remains an important consideration for choice of aneurysm repair. Open surgery is recommended in patients with connective tissue disease such as Marfan syndrome due to the unproved short and long term results with endovascular stent grafts. A recent consensus stated stent grafting in patients with Marfan syndrome or any other known connective tissue disorder is not recommended as there is limited information regarding the impact of persistent radial forces of a stent graft in the abnormal and weak aorta [27].

For the foreseeable future, it is likely that due to the various regulatory and device constraints associated with total endovascular repair, coupled with the absence of long-term outcome data, open TAAA repair will continue to be the gold standard treatment for thoracoabdominal aneurysms, especially in the younger population. Total endovascular repair is more attractive than hybrid repair and probably represents the future in patients unfit for open TAAA surgery. Open surgical repair has long-term outcome data, confers a lasting durable result and leads to good functional outcomes [28], which are all particularly important considerations in younger patients.

There are however limitations to consider with open surgical repair. Due to long-term durability, open repair has traditionally been offered to patients with a moderate life expectancy, and whilst replacement of the aneurysmal aortic segment with a synthetic graft has been the mainstay of treatment for over 40 years, the major disadvantage of open repair has been an associated increased 30-day mortality rate [9]-[12], and an increased length of hospital stay and rehabilitation over endovascular repair [29]. Other disadvantages include patch aneurysm formation in the long term. Whilst our 30 day mortality and hospital stay compare to other centres, one limitation of this study is we do not have data to look at long term outcomes for complications such as graft failure, patch aneurysm formation, and patency.

## Conclusions

In conclusion, contemporary open TAAA surgery performed in a specialist thoracoabdominal unit with careful pre-operative assessment, meticulous intra-operative technique and close post-operative support leads to good results in patients under the age of 60. It is against such results that emerging hybrid and endovascular techniques should be compared with due consideration given to the long-term risks. Endovascular techniques pioneered in the elderly and unfit may not represent the best long-term option for the younger patient with a thoraco-abdominal aneurysm.

## Consent

Written informed consent was obtained from the patients for the publication of this report and any accompanying images.

## Authors’ original submitted files for images

Below are the links to the authors’ original submitted files for images. Authors’ original file for figure 1 Authors’ original file for figure 2 Authors’ original file for figure 3 Authors’ original file for figure 4

## Abbreviations

TAAA: Thoracoabdominal aortic aneurysm
SEM: Standard error of mean
CTA: Computerised tomography angiography
SPSS: Statistical package for the social sciences
ROTEM: Rotational thromboelastometry
IQR: Interquartile range
RRT: Renal replacement therapy
LHB: Left heart bypass
COPD: Chronic obstructive pulmonary disease
