# Supplementary figures and images for: Contemporary outcomes of open repair of thoracoabdominal aortic aneurysm in young patients
Source: J Cardiothorac Surg. 2014 Dec 10;9:195. doi: 10.1186/s13019-014-0195-4 (PMC4269840; doi:10.1186/s13019-014-0195-4)

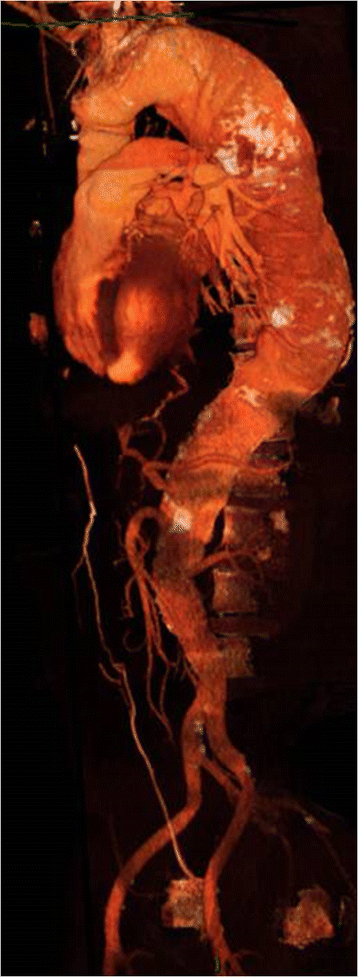

Supplement: Supplementary file 1 — Authors’ original file for figure 1 [file 13019_2014_195_MOESM1_ESM.gif]

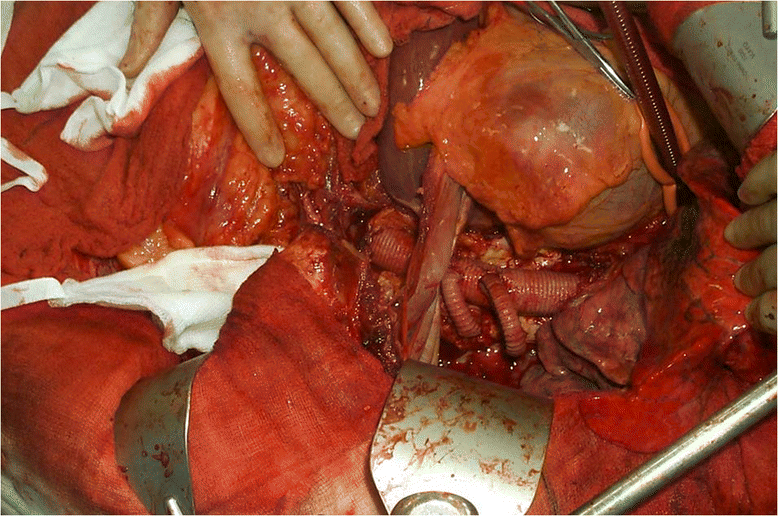

Supplement: Supplementary file 2 — Authors’ original file for figure 2 [file 13019_2014_195_MOESM2_ESM.gif]

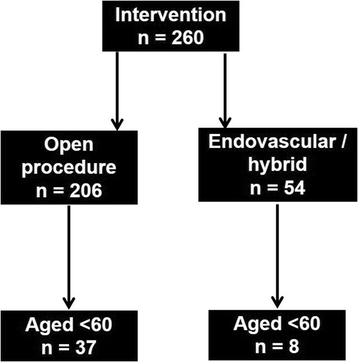

Supplement: Supplementary file 3 — Authors’ original file for figure 3 [file 13019_2014_195_MOESM3_ESM.gif]

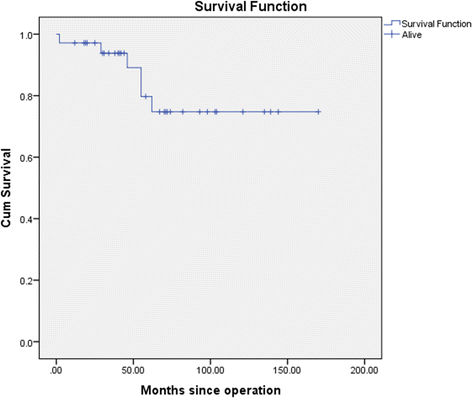

Supplement: Supplementary file 4 — Authors’ original file for figure 4 [file 13019_2014_195_MOESM4_ESM.gif]
